# Crafting Confidence: A Comprehensive Case Study of Full-Mouth Reconstruction Utilizing Implant-Supported Computer-Aided Design/Computer-Aided Manufacturing (CAD/CAM) Zirconia Restorations

**DOI:** 10.7759/cureus.65897

**Published:** 2024-07-31

**Authors:** Akansha Bansod, Sweta G Pisulkar, Arushi Beri, Utkarsh Umre, Ritul Jain, Shruti Deshmukh

**Affiliations:** 1 Prosthodontics, Sharad Pawar Dental College and Hospital, Datta Meghe Institute of Higher Education and Research, Wardha, IND; 2 Prosthodontics and Crown and Bridge, Sharad Pawar Dental College and Hospital, Datta Meghe Institute of Higher Education and Research, Wardha, IND; 3 Endodontics, Sharad Pawar Dental College and Hospital, Datta Meghe Institute of Higher Education and Research, Wardha, IND

**Keywords:** maxillofacial prosthodontics, geriatric rehabilitation, dental education, implantology, dental implants, patient care, full-mouth reconstruction, zirconia restorations, chronic periodontitis, cadcam

## Abstract

A 67-year-old male patient reported to the department with a chief complaint of tooth mobility. The patient presented with a medical history of diabetes for which he was on medication, and he also reported a history of chronic periodontitis. After a thorough assessment of the patient, a proper treatment plan was designed, which included full-mouth rehabilitation, prior to which the patient was advised full-mouth extraction. Six implants were inserted into the mandibular and maxillary arches as part of the treatment. After the implant was placed, zirconia was the preferred choice for the restoration because of its superior aesthetic results. Polyvinyl siloxane impression material was used to make implant impressions after an eight-week healing period. The case report provides the technique for the current approach to full-mouth rehabilitation with all six concepts of implant placement. In these situations, it is crucial to plan and insert implants correctly and adhere to the entire treatment plan. Restorations were performed using the latest computer-aided design/computer-aided manufacturing (CAD/CAM) technologies for a more aesthetically pleasing result. The case study highlights the significance of meticulous preparation and implementation for the accomplishment of successful full-mouth rehabilitation.

## Introduction

In past years, sub-periosteal implant-retained prostheses, either removable or fixed, and traditional complete denture therapy were the only options for restoring an edentulous dental arch [1-4]. Although sub-periosteal implants were linked to issues including mobility and dubious survival rates (approximately five to seven years), the complete denture had been the recommended line of action for treating the edentulous dental arch. An emerging method for restoring all of the missing teeth in an arch is the all-on-six implant treatment. Proper case selection is essential for the effective outcome of full-arch implant rehabilitation [5-10]. The surgical technique must be evaluated beforehand based on the radiographic and clinical findings, and prosthetic planning must be meticulous. This case series describes a variety of surgical and prosthetic techniques for implant-supported prosthetic rehabilitation of fully edentulous arches.

This case report describes a full-arch rehabilitation with six endosseous implants placed in each maxillary and mandibular arch, loaded according to the standard protocol. This rehabilitation utilized implant-supported zirconia restorations, which were made using computer-aided design/computer-aided manufacturing (CAD/CAM) technology.

## Case presentation

**Figure 1:**
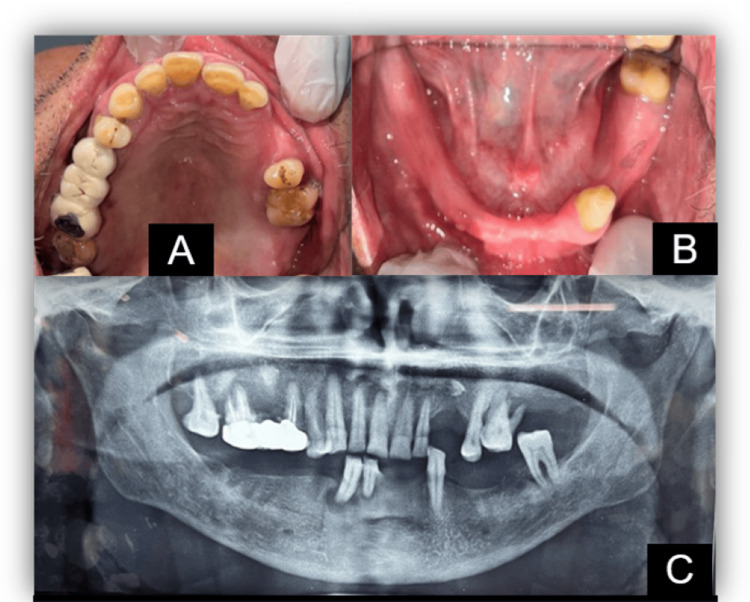
Preoperative records (A) Maxillary arch; (B) mandibular arch; (C) orthopantomograph

A 67-year-old male patient visited the dental outpatient department with several missing teeth and remaining teeth exhibiting mobility ranging from grade II to grade III, all displaying poor prognosis (Figure 1).

Medical history indicated the patient had been diagnosed with diabetes mellitus eight years prior and was currently under medication for the condition. Dissatisfied with the aesthetics and preferring not to have a removable prosthesis, the patient underwent a thorough evaluation. Subsequently, the full-mouth extractions were planned and implant-supported fixed prostheses for both the maxillary and mandibular arch rehabilitation were the optimal treatment planned.

Radiographic investigations

**Figure 2:**
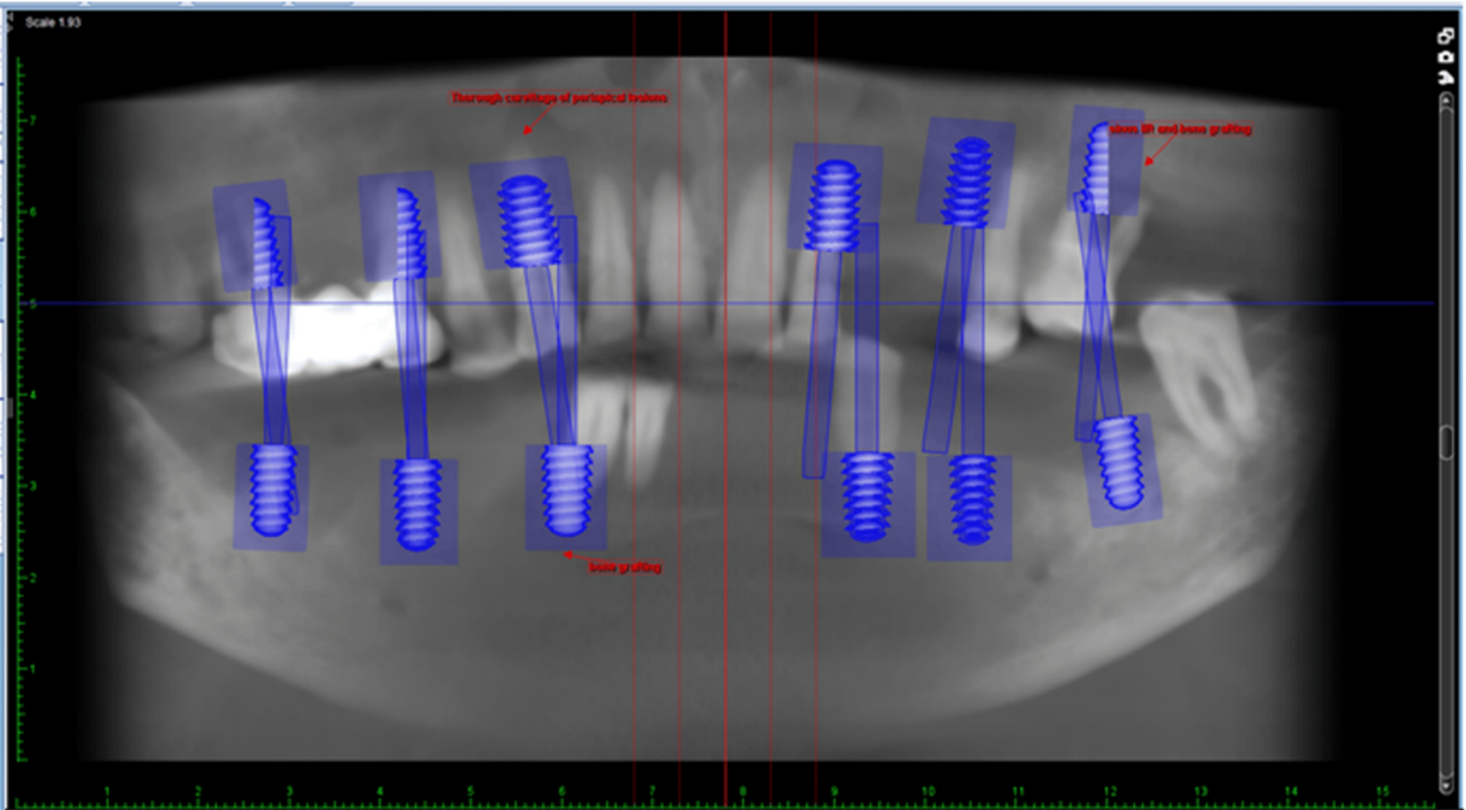
Proposed implant placement planning using Planmeca Romexis software (Helsinki, Finland)

Cone-beam computed tomography (CBCT) was the method of choice for the study. Figure 2 displays the information obtained from a CBCT scan of both arches.

Surgical phase

**Figure 3:**
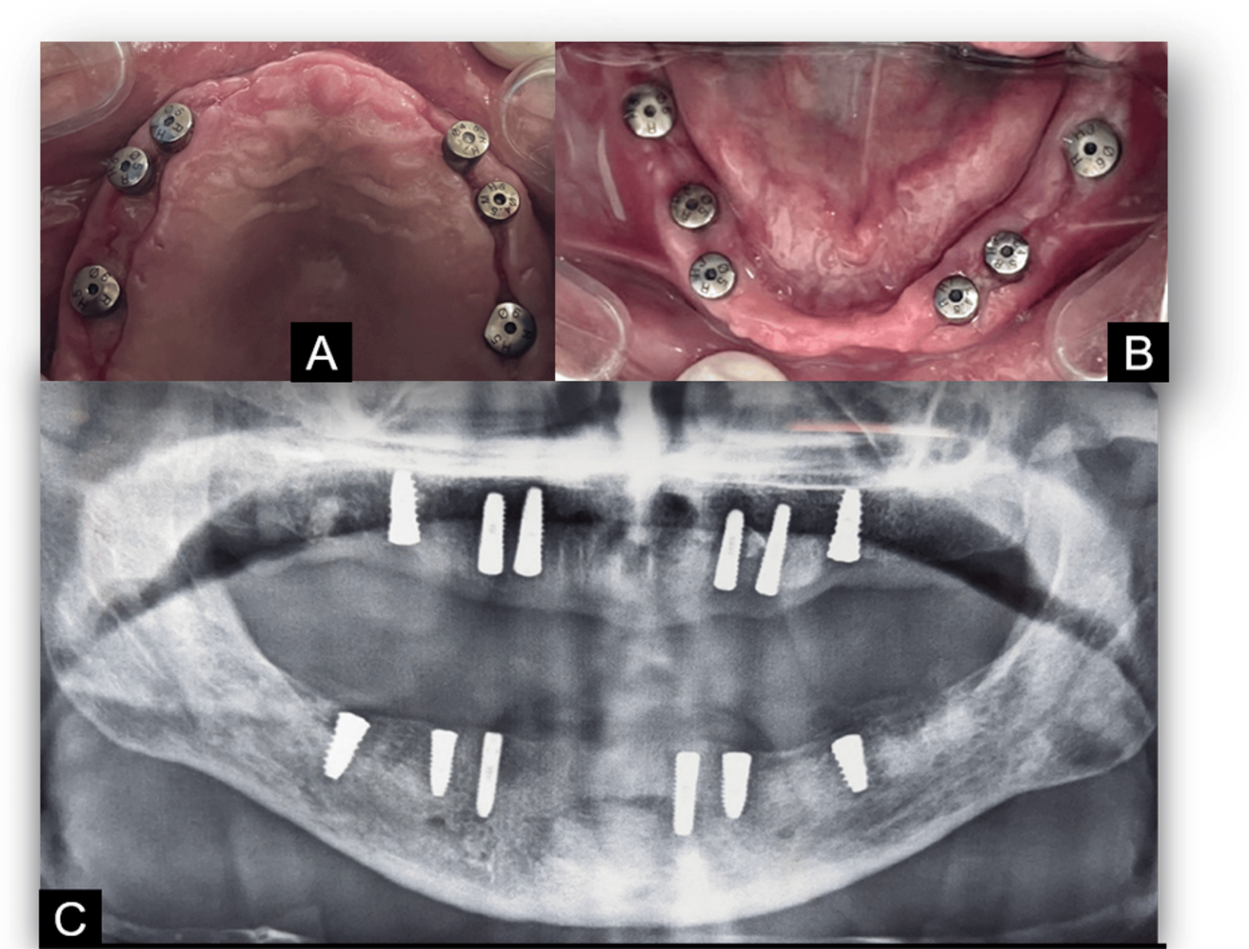
Postoperative images (A) Maxillary arch (six implants); (B) mandibular arch (six implants); (C) postoperative radiograph

Following a complete preoperative examination, lignocaine and 1:100,000 adrenaline were used to create a local anesthetic throughout the procedure. Extractions were done for all the remaining teeth. For all the 12 implants inserted, implant stability was adequate (35-40 N/cm as determined by a torque force). Figure 3 displays a combined total of 12 implants in both the maxilla and mandible.

Prosthetic phase

**Figure 4:**
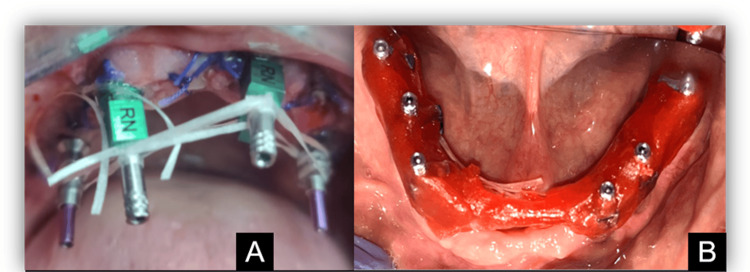
Splinting with floss and pattern resin

The healing abutments were removed from the implants, and open tray impression copings were chosen and fitted to the implants. These impression copings were splinted together using dental floss and pattern resin intraorally to provide increased stability and potentially greater precision (Figure 4).

**Figure 5:**
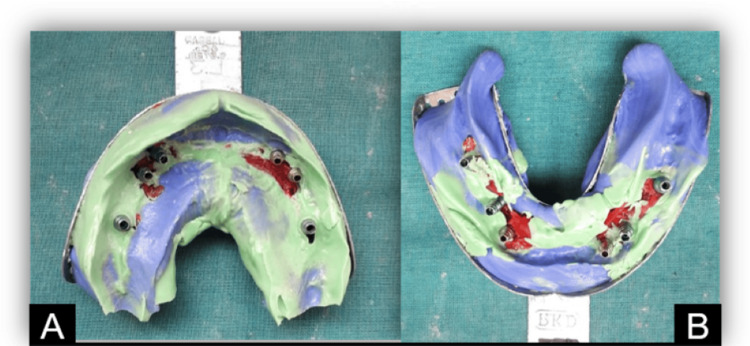
(A) Maxillary and (B) mandibular open tray impressions

The tray was positioned in the mouth with careful attention to ensure that the impression copings were aligned with the windows in an open tray. This allowed for easy retrieval of the impression copings while ensuring they were supported adequately by the impression material. The impressions of the implants were taken using the additional silicone of various viscosities (Figure 5).

**Figure 6:**
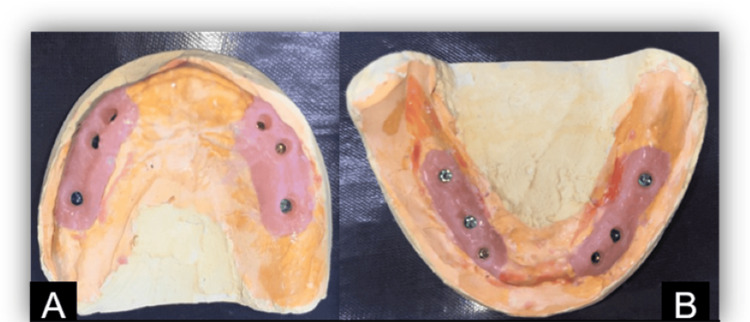
(A) Maxillary and (B) mandibular casts

After the impression was set, all of the impression copings were removed from the mouth along with the impression, and the cast was poured into die stone. The impression copings were unscrewed through the window on the tray (Figure 6).

**Figure 7:**
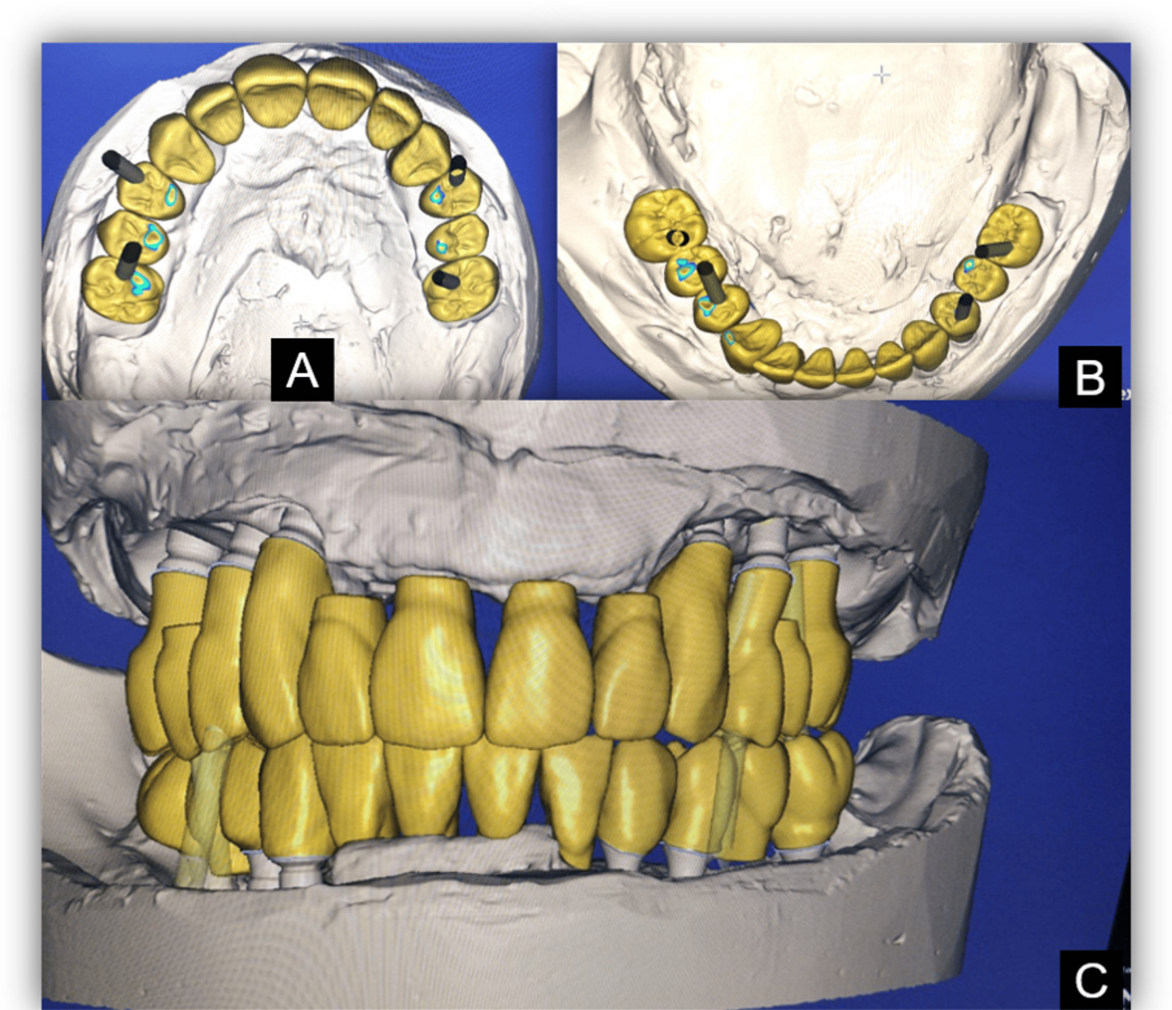
Computer-assisted designing using exocad software (A) Maxillary; (B) mandibular; (C) at occlusion

Using the wax occlusal rims, the vertical dimension at rest and occlusion (VDO) was measured using a divider, and the vertical dimension for occlusion was confirmed. A facebow record was established, and a centric relation record was made. Mounted casts were scanned using a lab scanner, and the design was completed using exocad software (Darmstadt, Germany) (Figure 7).

**Figure 8:**
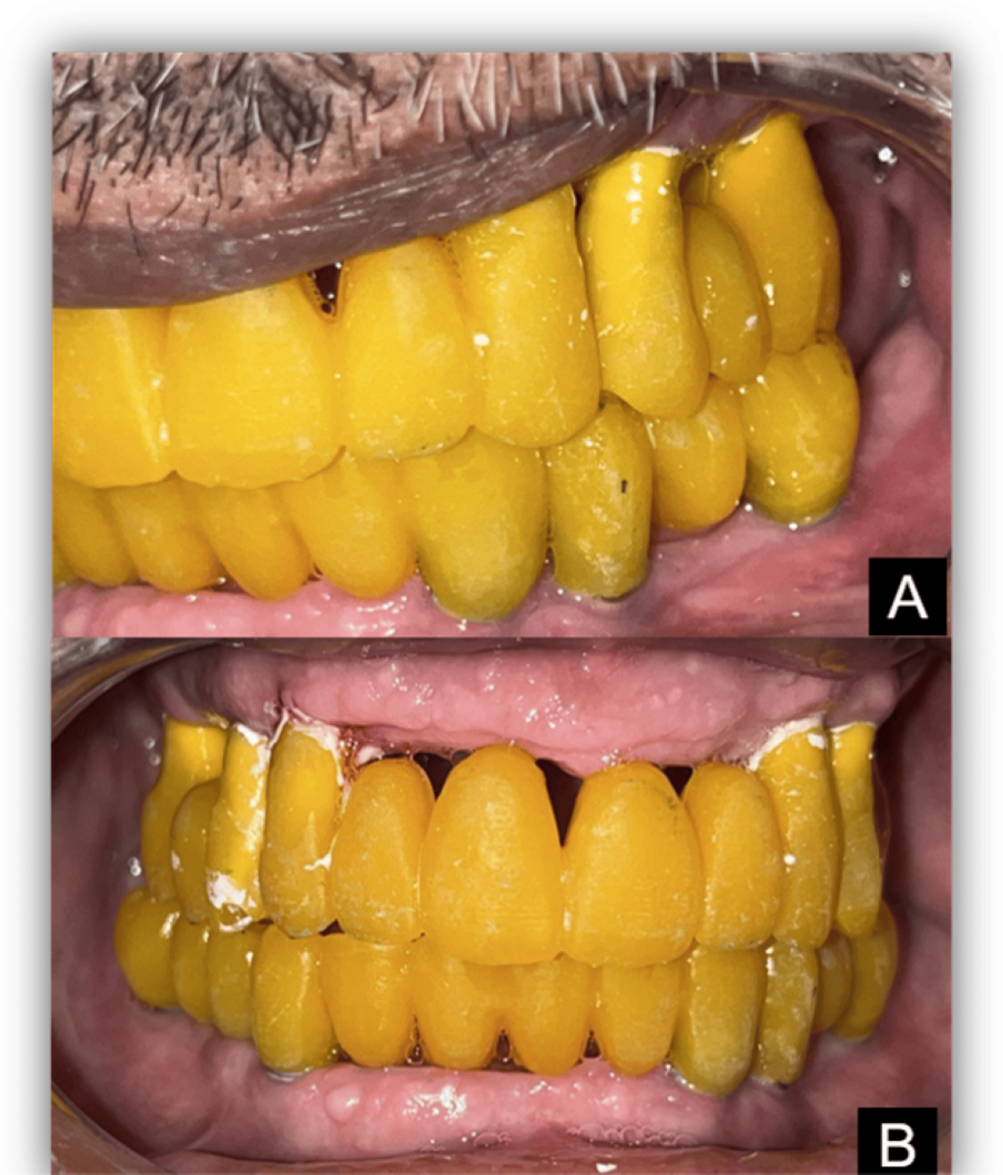
Verification of trial prostheses (A and B)

Following the assessment, milling was carried out. Trials were conducted, and occlusal adjustments were made (Figure 8).

The final restoration, fabricated in zirconia ceramic, took into consideration both aesthetic and functional aspects, focusing on several key factors: even distribution of centric contacts, anterior guidance for eccentric contacts distributed over multiple teeth, minimizing shear forces by maintaining shallow angles of tooth contact while still discluding the posterior teeth, achieving the centric relation position as defined by Dawson to regulate the tooth contacts, and adjusting the occlusal vertical dimension as needed to create proper incisal guidance and tooth form. Bite forces operating with these occlusal alterations mainly apply compressive forces to the prosthesis, implants, and bone.

**Figure 9:**
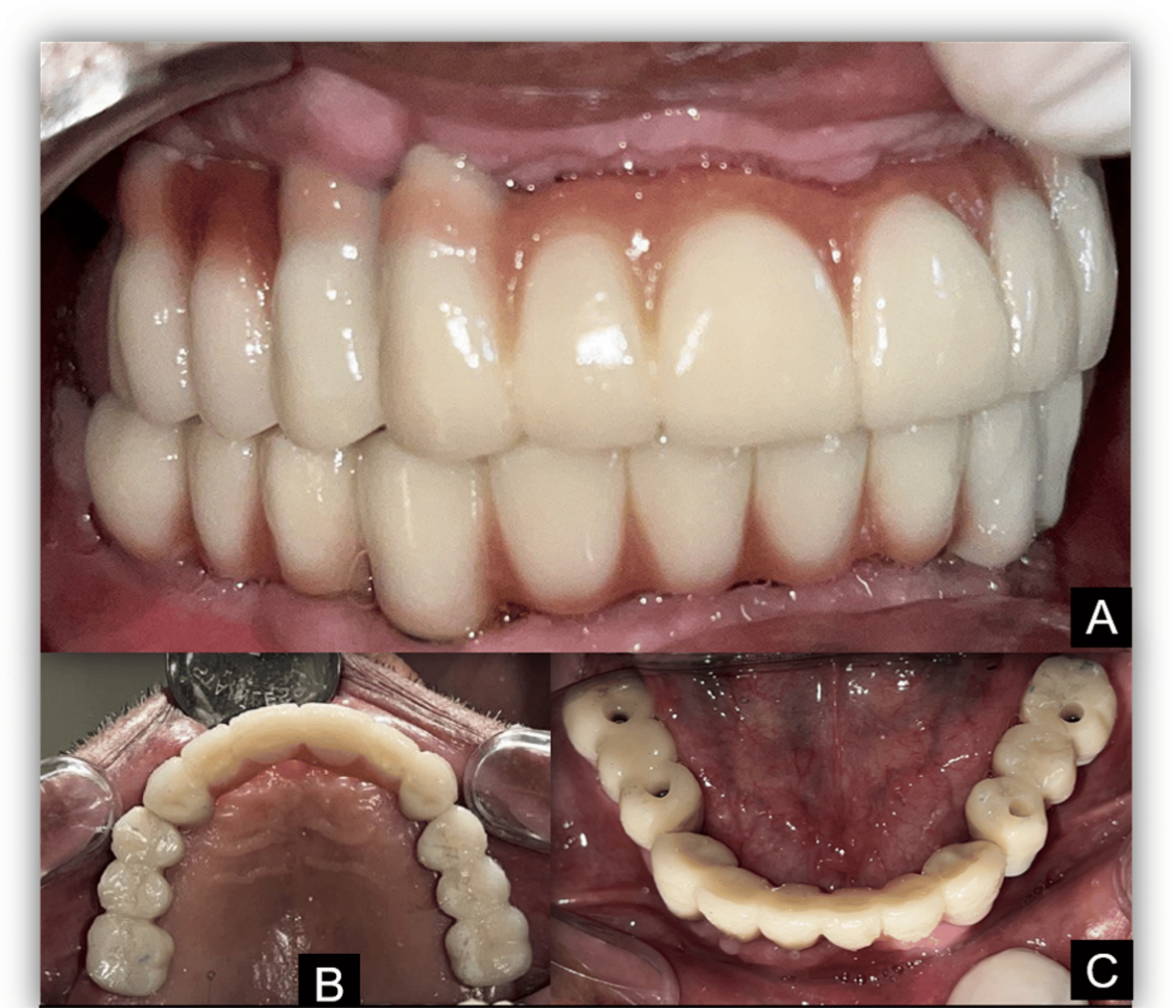
Zirconia implant-supported fixed dental prostheses (A) At occlusion; (B) maxillary arch; (C) mandibular arch

After milling, a bisque trial was conducted, and final adjustments were made before the prosthesis was screwed into place and the screws were covered with composite material (Figure 9).

**Figure 10:**
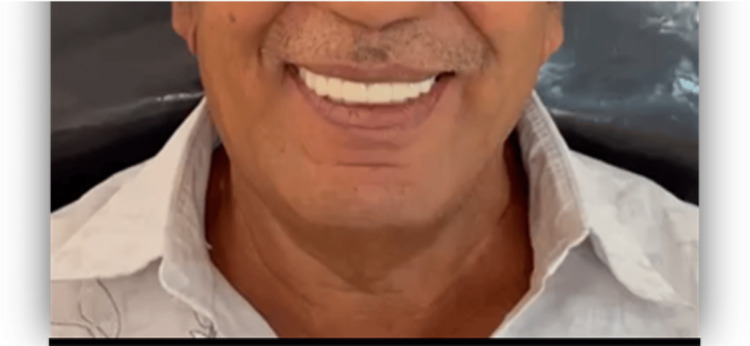
Post-prosthesis placement

**Figure 11:**
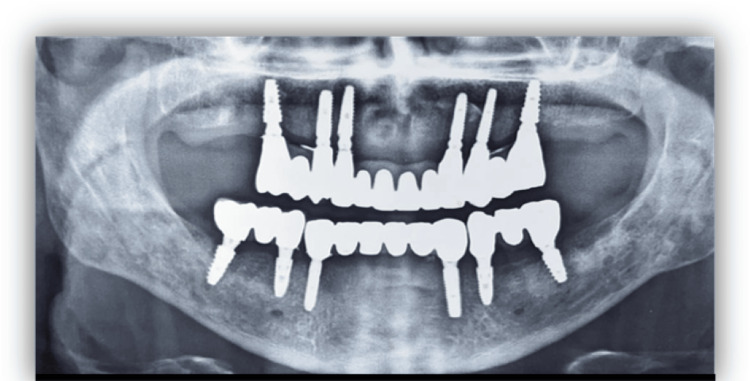
Postoperative radiograph

The clinical (Figure 10) and radiographic (Figure 11) image of the patient post-prostheses placement is as shown.

## Discussion

By establishing the idea of using four to six endosseous implants in the anterior part of the edentulous arch, i.e., the maxilla and mandible, to support a full-arch fixed prosthesis, Brånemark et al.'s (1977) work laid the groundwork for current dental implantology. Over a 10-year period, their study showed a good success rate, ranging from 78.3% to 80.3% for the maxilla and 88.4% to 93.2% for the mandible [11].

Our case study aimed to restore complete edentulism in the jaw using the all-on-six dental implant concept. Because of the remarkable stability and functionality of this method, the patient was able to regain his normal ability to chew and communicate. One of the main advantages of the all-on-six concept is that it can avoid unnecessary bone augmentation procedures, which minimizes surgical complexity and recuperation time. Additionally, the significant stabilization provided by the implants enables the fixed prosthesis to be attached quickly, yielding immediate functional and aesthetic benefits. Custom dentures that complement the patient's smile line and face structure improve long-term aesthetic satisfaction.

The present case study discusses the all-on-six treatment concept, although there have been comparisons between the all-on-four and all-on-six concepts for implant placement in the literature, and shows that, although the former may have advantages (such as lower costs and shorter treatment intervals), the all-on-six idea is better because it uses more implants and distributes stress more evenly. Research such as that conducted by Hassan et al. further demonstrated that the absence of cantilevers in the all-on-six approach reduces the risk of biomechanical problems [12-15]. Further research, such as that done by Agliardi et al., showed how important oral cleanliness is to the effectiveness of dental implants, as the all-on-six groups showed lower plaque levels than the all-on-four groups. This demonstrates how crucial meticulous prosthesis planning and maintenance are to achieving optimal outcomes [16].

The notion that an older patient's age limits the success of an implant is called into question by Park et al.'s data, which indicated that older patients could have successful implant placements with favorable long-term outcomes [17]. In our case, special thought was given to the smile line and transition zone during prosthesis planning in order to meet the patient's aesthetic criteria. Zirconia was chosen as the preferred material, and cautious occlusal correction ensured adequate bilateral occlusion.

When compared to traditional procedures, the all-on-six concept is a significant advancement in dental implantology that yields better outcomes in terms of stability, practicality, and aesthetics. However, particular patient characteristics and meticulous prosthetic planning are still required for long-term success.

## Conclusions

For patients who are edentulous or have teeth with a poor prognosis, full-mouth dental implant rehabilitation offers a treatment that combines stability, functionality, and aesthetic satisfaction. A full-mouth dental implant is a major advancement in modern dental implantology. Six dental implants are used in this surgery, which provides excellent support for a fixed prosthesis, allowing an improved aesthetic for the patient, which overall improves the patient's quality of life. The following case study demonstrates the significant advantages of the all-on-six implant concept, including its ability to reduce the need for additional bone augmentation procedures, which reduces surgical complexity and expedites healing. The restoration process helps patients restore confidence and enhances their general quality of life by allowing them to observe changes in appearance and function instantly.

The all-on-six dental implant concept represents a substantial improvement in the field of dental restoration as it provides patients with a comprehensive solution that takes into account both empirical and aesthetic factors. The all-on-six concept has the potential to significantly enhance patient outcomes for patients with complete edentulism when paired with adherence to implantology best practices and careful evaluation of patient circumstances.
